# Rapid Detection of Isoniazid Resistance in *Mycobacterium tuberculosis* by a Single Multiplex Allele-specific Polymerase Chain Reaction Assay

**Published:** 2011-08-24

**Authors:** Kimia Taghavi, Parissa Farnia, Mohammad Varahram, Fatemeh Maryam Sheikhoslami, Mojtaba Ahmadi, Mehdi Kazempoor, Mohammad Reza Masjedi, Ali Akbar Velayati

**Affiliations:** 1. Mycobacteriology Research Centre, Shahid Beheshti Medical University, Tehran, Iran; 2. Statistics Deptartment, Mycobacteriology Research Centre, Shahid Beheshti Medical University, Tehran, Iran; 3. Internal Medicine Department, Mycobacteriology Research Centre Shahid Beheshti Medical University, Tehran, Iran; 4. Pediatric Deptartment, Mycobacteriology Research Centre, Shahid Beheshti Medical University, Tehran, Iran

**Keywords:** Multidrug Resistant, Tuberculosis, PCR

## Abstract

**Objective:**

Global surveillance has shown that drug resistant (DR) tuberculosis (TB) is widespread. Prompt detection of *Mycobacterium tuberculosis* drug resistance is essential for effective control of TB. The most frequent mutations associated with Isoniazid (INH) resistance in Mycobacterium are substitutions at codons 315 in the *katG* gene and the *mabA-inhA* promoter region (−15). This survey evaluated INH resistant-associated mutations in order to determine rapid detection of TB resistance.

**Materials and Methods:**

Through a descriptive cross- sectional study total of 96 sputum specimens were digested, examined microscopically for acid-fast bacilli and inoculated into Löwenstein-Jensen slants. Thereafter, the susceptibility and identification tests were performed on culture positive specimens. Subsequently, the strains were subjected to multiplex allele-specific polymerase chain reaction (MAS-PCR) targeting in the codons 315 in the *katG* gene and the *mabA-inhA* promoter region. Distinct PCR banding patterns were observed for different mutation profiles.

**Results:**

Drug susceptibility testing revealed that out of 96 available isolates, 30 (31.3%) were susceptible, 36 (37.5%) had multi-drug resistance (MDR-TB) and 30 (31.3%) showed mono- drug resistance. In comparison with the culture-based phenotypic drug susceptibility test, the sensitivity and specificity of MAS-PCR assay for drug resistance-related genetic mutations were 76.7% and 71.4%, respectively. The correlation between MAS-PCR and culture-based phenotypic drug susceptibility testing findings was 99.4%.

**Conclusion:**

The profile of the isolates suggests a significant number of different DR strains with a high frequency of mutations at codon 315 of the *katG* gene. MAS-PCR provides a rapid, simple and cost-effective method for detecting MDR-TB.

## introduction

**Fig 1:**
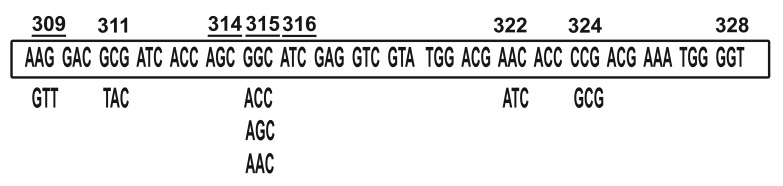
Location, type, frequency of mutations in the kat**G** gene located in region **309-328** bp (**29** strains of **M**. tuberculosis).

Tuberculosis (TB) is a growing international health concern. It is the biggest killer among the infectious diseases in the world despite the use of a live attenuated vaccine and several antibiotics (1, 2). According to the World Health Organization, the estimated incidence of TB in Iran was 28 cases per 100 000 in the year 2008 (3). Iran's TB prevalence has declined by about 8.7% during the past 30 years (4). TB prevalence was 1.3% in 2008. Iran reported 9423 new TB cases, which comprised about 56% of the country's total TB cases in 2008 (3). A total of 80% of TB patients in Iran are of ages 15 to 49 years (5). Recent data has documented increased numbers of mono-drug resistance among new TB cases within the country in many regions (1, 6). Tehran is one of the oblast (important region) and the capital of Iran. Many people travel to Tehran from other countries and other cities of Iran [All endemic regions of Iran; Zabol (Afghanistan border); Gorgan (Turkmenistan border); Tabriz (Azerbaijan border) and Iraq] with active pulmonary tuberculosis. Iran's border is an endemic region in Asia with 10-13% multiple drug resistant (MDR) among 141 TB cases per 100,000 (5). Isolation, identification and drug susceptibility testing of TB and other clinically important mycobacteria can take several weeks because of its slow growth rate (1, 7, 8). Phenotypic drug susceptibility testing takes approximately two months (9). Early detection of drug resistance allows the initiation of appropriate treatment, which has an impact on better disease control (9, 10). The development of Isoniazid (INH) resistance usually precedes resistance to rifampicin (RF), therefore resistance to INH is considered as a surrogate marker for MDR-TB (11). Considering the severity of diseases associated with the spread and transmission of MDR or strains, extremely drug resistant ruberculosis (XDR-TB), we attempted to develop a rapid method to determine TB drug resistance. Resistance to multiple drugs is the consequence of an accumulation of mutations (7, 12) (Fig 1). The most frequent mutations associated with INH resistance in Mycobacterium are the substitutions at codons 315 in the *katG* gene and mabA-inhA in the promoter region -15.

The *katG* gene encodes mycobacterial catalase peroxidase, which is the only enzyme in TB capable of activating the pro-drug INH to its active form (13). Furthermore, the *katG* gene is involved in detoxification of endogenously generated or exogenously supplied hydrogen peroxide. Hence, the aim of this study was to characterize these mutations in Mycobacterium isolates in new and previously treated TB cases suspected to be infected with drug-resistant (DR) pulmonary TB by a multiplex allele-specific polymerase chain reaction (MAS-PCR) that detects INH resistance-associated genetic mutations at a lower cost, than the currently available methods.

## Materials and Methods

### Data Collection

This was a descriptive cross- sectional study conducted from December 2008 to July 2009. A total of 96 pulmonary TB patients hospitalized at the Masih Daneshvari Hospital in Tehran, Iran were recruited. The identification of TB complex strains was based on conventional methods, including the niacin production test, the nitrate reduction test, optimum temperature and time for growth, pigment production and colony morphology (14).

Case data were collected by trained technicians using standard questionnaires. Informed written consents were taken from all patients. Information was obtained on sex, age, contact (family contact/ close contact), previous TB history, present address and associated medical data such as human immunodeficiency virus (HIV) infection (yes, no, not known), and tuberculin skin test (+, -, equivocal). The study was approved by the Ethics Committee of Shahid Beheshti Medical University.

### Bacterial isolation

Primary isolation and culturing of Mycobacterium from sputum specimens were followed in accordance to procedures manual (11). Drug susceptibility testing against INH, RF, streptomycin (SM), ethambutol (ETB) and pyrazinamide (PZA) were performed by the proportional method on Löwenstein- Jensen media at concentrations of 0.2, 0.4 and 2.0 µg/ml, respectively. Drug resistance was defined as greater than 1% growth in the presence of 0.1 µg of INH per milliliter (14, 15).

### DNA extraction and MAS-PCR

Genomic DNA was extracted as described by the Cetyl Trimethylammonium Bromide Procedures Manual (8, 11). Quality of extracted DNA was measured using the Picopet01 DNA Calculator (Cambridge, England). To evaluate MAS-PCR assay, two allele-specific primers were utilized based on a previous study by Yang et al. (11). These primers corresponded to the two codons where most point mutations have been found according to wild-type sequences of strain H37RV (ATCC35827). For each MAS-PCR reaction, a standard 50 µL reaction mixture was used. Each reaction mix included four primers: katGOF (10 pmol in 1 µL), katG5R (10 pmol in 1 µL), inhAP- 15 (10 pmol in 1 µL) and inhAPF2 (10 pmol in 1 µL). The other reagents included in each reaction mix were: 2 µL of 50× deoxyribonucleotide mix (0.2 mM of each deoxyribonucleotide), 5 µL of 10× reaction buffer, 1 unit of AT taq hot star DNA polymerase (Vivantis, London, England) and 100- 200 ng of DNA template. Primer sequences are mentioned in table 1. The thermocycling program included an initial denaturing at 96℃ for 5 minutes, 40 cycles of 96℃ for 30 seconds, 68℃ for 30 seconds, 72℃ for 30 seconds and a final extension at 72℃ for 7 minutes.

### DNA sequencing

To gain an understanding of the INH-associated genetic mutations, we sent isolates to the Super National Institute of TB (Sweden) to perform complete DNA sequencing of the involved genes.

**Table 1:** Primers for MAS-PCR to detect INH resistance mutations of M. tuberculosis.


Detection targets	Allele-specific primers (5'-3')	Paired primers	Length of PCR product (bp)
katG315	katG5Ra	ATACGACCTCGATGCCGC	292
mabA-inhA: -15	InhAP-15	GCGCGGTCAGTTCCACA	270


The entire *inhA* structure gene and a 648-bp long *mabA-inhA* promoter region that extended from 271 bp upstream of the *mabA* gene to 377 bp of the adjacent *mabA* structure gene were sequenced by primers katGIF (AGCTCGTATGGCACCGGAAC) and katG4RB (AACGGGTCCGGGATGGTG), and Qiagen taq PCR Master. The PCR products for DNA sequencing were purified using the QIAquick PCR Purification Kit according to the instructions of the manufacturer (QIAGEN, Stanford, Valencia, CA) and then sent to Sweden for sequencing.

The sequence of TB H37Rv was used as the reference for sequence comparison.

### Phenotypic test quality controls

Phenotypic test quality controls were based on conventional methods, including the niacin production test, the nitrate reduction test, optimum temperature and time for growth, pigment production and colony morphology (1, 7).

### Statistical analysis

Continuous variables were expressed as group means ±SD. Results of non-parametric findings were analyzed by Mann Whitney and Kruskal Wallis tests. P≤0.05 was considered significant.

## Results

Specimens collected from 96 patients were sorted into two different regions of Iran: Tehran as the capital (56 isolates) and other cities (33 isolates). Additionally, there were isolates obtained from immigrant patients from Iraq (6 isolates) and Russia (1 isolate). The prevalence of drug resistance to at least one anti-TB drug was 70% (21 isolates) in Tehran and 30% (9 isolates) in other regions. The prevalence of MDR was 36.11% (13 isolates) in Tehran, 44.44% (16 isolates) from other regions, 16.66% (6 isolates) from Iraq and 2.7% (1 isolate) from Russia. The prevalence of drug sensitive patients was 73.33% (22 isolates) in Tehran and 26.66% (8 isolates) from other regions. The median age was 50 years (p=0.000). Fifty-three (55.2%) were male and 43 (44.8%) were female. The male to female ratio was 1.2:1. Based on the phenotypic drug susceptibility testing results, 56 cases (58.3%) were resistant to INH. There were 36 patients (37.5%) who were MDR-TB cases and 30 patients (31.3%) had mono DR strains, of which 20 (66.6%) were INH-resistant. Based on multiplex PCR, 43 isolates showed resistance to INH, of which 16 belonged to the mono DR group, 26 belonged to the MDR group and 1 was from the susceptible group. Forty-one (62.1%) resistant isolates showed mutations in katG315 and 6 (9%) exhibited mutations at the mabA-inhA promoter region -15. Details are provided in table 2.

**Fig 2:**
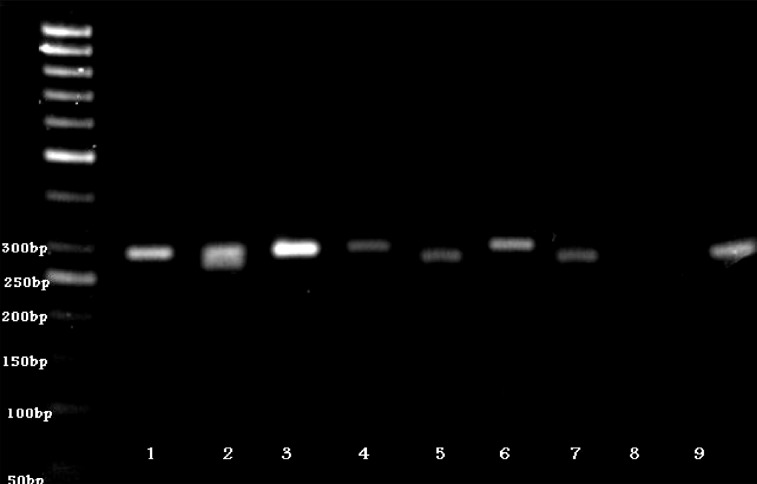
Results of **MAS-PCR** shown by 8% polyacrylamide gel electrophoresis The 292-bp band represents the *katG* codon 315-specific **PCR** product and the 270-bp band represents the -15 promoter region of the mabA-inhA specific **PCR** product. In case of a mutation existing in a given codon or region, no related allele-specific **PCR** product was generated. Lane 2 represents the reference strain **H37Rv;** lane 7 represents the negative control. The remaining lanes represent isolates that were found to have point mutation(s) at the targeted loci. Sites with mutations in each of the remaining lanes are as follows. Lanes 5, 8 and 9: *katG* codon 315; lanes 1,3,4 and 6: mabA-inhA.

PCR products were visualized by 8% polyacrylamide gel electrophoresis (Fig 2). The 292-bp band represented the *katG* codon 315-specific PCR product and the 270-bp band represented the -15 promoter region of the mabA-inhA-specific PCR product.

**Table 2:** Study group sex and nationality data


Group		MDR		Non-MDR			Susceptible		Total	
		Count	N (%)	Count	N (%)	Count	N (%)	Count	N (%)
Sex	Female	18	50.0	11	36.7	14	46.7	43	44.8
	Male	18	50.0	19	63.3	16	53.3	53	55.2
	Total	36	100.0	30	100.0	30	100.0	96	100.0

Nationality	Iranian	20	55.6	26	86.7	20	66.7	66	68.8
	Afghan	5	13.9	4	13.3	10	33.3	19	19.8
	Baqu	3	8.3	0	0.0	0	0.0	3	3.1
	Iraqi	8	22.2	0	0.0	0	0.0	8	8.3
	Total	36	100.0	30	100.0	30	100.0	96	100.0


Thus, when no mutation existed at the targeted codon, the wild-type allele-specific fragment was amplified and when there was a mutation at the targeted codons, no allele-specific PCR product was generated.

Ninty-six percent of susceptible isolates showed the expected wild-type allele-specific patterns however one (4%) of the susceptible isolates had the katG315 mutation, which was confirmed by repeating the assay and DNA sequencing (Table 3).

Direct sequencing of the *katG* gene revealed a point mutation in 24 out of 26 (92.3%) MDR specimens (shown in molecular method) and the remaining 2 (7.7%) had wild type *katG* (no evidence of mutation) which was statistically significant (p<0.001). A point mutation was found in 24 out of 24 (100%) isolates with the serine --->threonine substitution (AGC-->ACC) ( Table 4). Using the results of the culture- based phenotypic drug susceptibility testing as the reference standard, we assessed the sensitivity and specificity of MAS-PCR for detection of INH resistance. The correlation between the proportional method and MAS-PCR were determined to evaluate the usefulness of MAS-PCR in determining genetic mutations in clinical isolates.

**Table 3:** Results of **MAS-PCR** in comparison to the phenotypic method to detect **INH**-resistant mutations of **M.** tuberculosis


	Group	Result	Percentage
	MDR	36	37.4%
	non-MDR	30	31.3%
	Sensitive	30	31.3%
Phenotypic	Resistant	56	58.3%
Drug test	Susceptible	40	41.7%
Molecular	Resistant	42	43.8%
Drug test	Susceptible	54	56.3%
*katG315*	No mutation	54	56.3%
Mutation	Mutation	42	43.8%
*inhA*	No mutation	90	93.8%
Mutation	Mutation	6	6.3%


The sensitivity and specificity of the MAS-PCR for detecting INH resistance were assessed using culture results as a reference. The sensitivity and specificity of the MAS-PCR assay were 76.7% and 71.4% respectively (Table 5).

**Table 4:** Results of **MAS-PCR** in comparison to the phenotypic method


Cross-tabulation					
			INH molecular method		Total
			Resistant	Susceptible	
INH phenotypic method	Resistant	Count	41	15	56
		% of total	42.7%	15.6%	58.3%
	Susceptible	Count	1	39	40
		% of total	1.0%	40.6%	41.7%
Total		Count	42	54	96
		% of total	43.8%	56.2%	100.0%


**Table 5:** Sensitivity and specificity of **MAS-PCR** assay and phenotypic drug susceptibility testing for detecting **M.** tuberculosis resistance to **INH** among 96 clinical isolates


MAS-PCR results	Culture results		Sensitivity (%)	Specificity (%)
	No. resistant	No. susceptible		
INH mutation Detected	43 (56)	53 (40)	76.7%	71.4%


## Discussion

According to some studies, INH resistance dependant to the katG315 codon mutation has also been identified as a predominant TB problem in Iran (3, 15). Our results correlate with this finding. Our data is in concordance with those reported in Sweden by Pontus (13) in Northwestern Russia by Mokrousov et al. (8). As shown in other studies, the resistance to INH drug among TB patients in Iran occurred with a significant prevalence (9, 16). On the contrary, in Iran a high prevalence of mutations in codon 315 was detected. The researchers examined 54 INH-resistant isolates and found 100% *katG* point mutations, whereas only 7.5% had a codon 315 substitution; the remaining mutation positions were at codons other than 315 (2). In addition, Pontus found that mutations in codon 315 were detected in 18 (64%) out of 28 INH-resistant isolates from Dubai and in 6 (35%) out of 17 resistant strains from Beirut (13). Our results supported the hypothesis of linking the *katG* gene mutation to the development of INH-resistance in MTB. Taken together, we found a 99.4% correlation between culture-based phenotypic drug susceptibility, sequence testing and MAS-PCR. In our study, 13 isolates (14/7%) were not detected by the MAS-PCR method (Table 2). The absence of mutations in 7.7% of resistant isolates could be attributed to possible involvement of other codon positions at the same gene or other genes, rather than the studied *katG*. A major limitation to molecular genetic detection of drug resistance by any technique is that molecular genetic tests detect only known mutations. This may be due to a different prevalence of mutations that may be geographically related. Another explanation could be the so-called "heteroresistance", which means the presence of a mixture of susceptible and resistant subpopulations in a culture which could be an obstacle against the sensitivity of molecular drug resistance testing and successive therapy (17). Overall, MAS-PCR is less technically demanding, simple, reliable and requires less time and less expensive equipment, which makes it more accessible for resource-limited countries such as Iran. This rapid drug susceptibility test has proven to be cost effective, as well as shown to allow for more rapid treatment and consequently reduce the development of resistance.

## Conclusion

The DR profile of the isolates suggests a significant number of different DR TB strains with a high frequency of mutations at codon katG315. This result leads us to consider different regions of the same genes, as well as other genes for further analysis, which is important if a geneticbased diagnosis of DR-TB is to be developed for this region. Although the MAS-PCR, as with any other molecular method, cannot yet completely replace the culture-based phenotypic susceptibility test because of the limitation mentioned above, it provides a rapid screening tool for a majority of DR isolates. If implemented, this assay would help clinicians directly prescribe an effective treatment and be a tool for the prevention of MDR and XDR-TB within the country.

## References

[1] Farnia P, Masjedi MR, Varahram M, Mirsaeidi M, Ahmadi M, Tabarsi P (2008). The Recent-Transmission of
Mycobacterium tuberculosis Strains among Iranian
and Afghan Relapse Cases: a DNA-fingerprinting using
RFLP and spoligotyping. BMC Infect Dis.

[2] Zaker Bostanabad S, Velayati AA, Masjedi MR, Titov LP, Taghikhani M, Khazaei HA (2006). katG mutation of
isoniazid-resistant isolated from tuberculosis patients. Tanaffos.

[3] Lankarani Baqeri K (2009). Decrease Tuberculosis outbreak
in Iran, eng. Farsnews.

[4] World Health Organization (2005). Stop TB partnership annual
report 2004.

[5] Tavakoli A, Safaee HG, Navvabakbar F, Salehi M, Bahremand A, Nasr Isfahani (2005). Mutations in the proB
Gene of rifampin resistant Myco- bacterium tuberculosis
Isolated in Isfahan by PCR-SSCP. J Sci.

[6] Namaei M, Sadeghian A, Naderinasab M, Ziaee M (2006). Prevalence of primary drug resistant Mycobacterium
tuberculosis in Mashhad, Iran. Indian J Med Res.

[7] Masjedi MR, Farnia P, Soroosh S, Pooramiri MV, Mansoori SD, Zarifi AZ (2006). Extensively drug-resistant tuberculosis:
2 years of surveillance in Iran. Clin Infect Dis.

[8] Mokrousov I, Otten T, Vyshnevskiy B, Narvskaya o (2003). Allele-specific rpoB PCR assays for detection of
rifampin-resistant Mycobacterium tuberculosis in sputum
smears. Antimicrob Agents Chemother.

[9] Khalilzadeh S, Boloorsaz MR, Safavi A, Farnia P, Velayati AA (2006). Primary and acquired drug resistance in
childhood tuberculosis. East Mediterr Health J.

[10] Johnson R, Streicher EM, Louw GE, Warren RM, van Helden PD, Victor TC (2006). Drug resistance in Mycobacterium
tuberculosis. curr Issues Mol Biol.

[11] Yang Z, Durmaz R, Yang D, Gunal S, Zhang L, Foxman B (2005). Simultaneous detection of isoniazid,
rifampin, and ethambutol resistance of Mycobacterium
tuberculosis by a single multiplex allele-specific polymerase chain
reaction (PCR) assay. Diagn Microbiol Infect Dis.

[12] Baghaei P, Tabarsi P, Chitsaz E, Saleh M, Marjani M, Shemirani S (2010). Incidence, clinical and epidemiological
risk factors, and outcome of drug-induced hepatitis
due to antituberculous agents in new tuberculosis
cases. Am J Ther.

[13] Pontus J (2008). Molecular characterisation of antibiotic resistance
in Mycobacterium tuberculosis.

[14] Merza MA, Farnia P, Salih AM, Masjedia MR, Velayatia AA (2010). The most predominant spoligopatterns of
myco-bacterium tuberculosis isolates among Iranian,
Afghan-Immigrant, Pakistani and Turkish tuberculosis
patients: a comparative analysis. Chemotherapy.

[15] Velayati AA, Farnia P, Masjedi MR, Ibrahim TA, Tabarsi P, Haroun RZ (2009). Totally drug-resistant tuberculosis
strains: evidence of adaptation at the cellular level. Eur Respir J.

[16] Zaker S, Bahrmand AR, Poorazar SH, Abdolrahimi F, Nur-Nemattollahi A (2007). Mutations in codon 315 of the
katG gene associated with high-level resistance to isoniazid. Tanaffos.

[17] Farnia P, Masjedi MR, Nasiri B, Mirsaedi M, Sorooch S, Kazeampour M (2007). Instability of IS6110 patterns
in multidrug-resistant strains of Mycobacterium tuberculosis. Epidemiol Infect.

